# Colorectal cancer genomics: evidence for multiple genotypes which influence survival

**DOI:** 10.1054/bjoc.2001.2095

**Published:** 2001-11

**Authors:** P H Rooney, A Boonsong, J A McKay, S Marsh, D A J Stevenson, G I Murray, S Curran, N E Haites, J Cassidy, H L McLeod

**Affiliations:** Departments of Medicine and Therapeutics, Medical Genetics, Pathology, University of Aberdeen, Foresterhill, AB25 2ZD, Aberdeen

**Keywords:** CGH, colo-rectal tumours, cell lines, survival, cancer genomics

## Abstract

Colorectal cancer (CRC) is a leading cause of cancer death and the mechanism for variable outcome in this disease is not yet fully understood. It is hypothesized that differences in the genetic make-up of tumours may be partially responsible for the differences observed in survival among same staged individuals for this disease. In this study the tumour genomes of 29 consecutive patients undergoing surgery for Dukes' C CRC were assessed by comparative genomic hybridization (CGH). In addition, the CGH profiles from the tumours were compared with those from eight colorectal cell lines. Great variation in genetic grade (all detectable aberrations i.e., loss + gain) was observed in 29 Dukes' C colorectal tumours by CGH (median four aberrations per tumour, range 0–20). Gain was found in 76% and loss in 41% of tumours. The most frequently observed regions of gain were 13q (27.6%), 20q (27.6%), 7p (24.1%), 8q (24.1%), and 1q (20.7%) and loss were 18q (31%), 4q (20.7%), 17p (20.7%), 18p (20.7%), and 15q (20.1%). None of these specific genomic aberrations were associated with patient survival. However, patients with more than two aberrations had a better survival than patients with fewer regions of loss and gain (*P* = 0.02). CRC cell lines had similar regions of loss or gain as the tumours. However, the frequency of genomic aberrations was much greater in the CRC cell lines. Although genomic change in CRC is relevant to the survival of patients with Dukes' C CRC, careful analysis is required to identify cell lines which are representative models of CRC genomics.© 2001 Cancer Research Campaign  http://www.bjcancer.com


British Journal of Cancer (2001) 85(10), 1492–1498
© 2001 Cancer Research Campaign
doi: 10.1054/ bjoc.2001.2095, available online at http://www.idealibrary.com on IDEAL

### Colorectal cancer genomics: evidence for multiple genotypes which influence survival

**PH Rooney[1], A Boonsong[1], JA McKay[1], S Marsh[1], DAJ Stevenson[2], GI Murray[3], S Curran[3], NE Haites[1,2], J Cassidy[1] and HL McLeod[1]**

Departments of [1]Medicine and Therapeutics; [2]Medical Genetics; and [3]Pathology, University of Aberdeen, Foresterhill, Aberdeen AB25 2ZD

**Summary** Colorectal cancer (CRC) is a leading cause of cancer death and the mechanism for variable outcome in this disease is not yet fully understood. It is hypothesized that differences in the genetic make-up of tumours may be partially responsible for the differences observed in survival among same staged individuals for this disease. In this study the tumour genomes of 29 consecutive patients undergoing surgery for Dukes' C CRC were assessed by comparative genomic hybridization (CGH). In addition, the CGH profiles from the tumours were compared with those from eight colorectal cell lines. Great variation in genetic grade (all detectable aberrations i.e., loss + gain) was observed in 29 Dukes' C colorectal tumours by CGH (median four aberrations per tumour, range 0–20). Gain was found in 76% and loss in 41% of tumours. The most frequently observed regions of gain were 13q (27.6%), 20q (27.6%), 7p (24.1%), 8q (24.1%), and 1q (20.7%) and loss were 18q (31%), 4q (20.7%), 17p (20.7%), 18p (20.7%), and 15q (20.1%). None of these specific genomic aberrations were associated with patient survival. However, patients with more than two aberrations had a better survival than patients with fewer regions of loss and gain ($P = 0.02$). CRC cell lines had similar regions of loss or gain as the tumours. However, the frequency of genomic aberrations was much greater in the CRC cell lines. Although genomic change in CRC is relevant to the survival of patients with Dukes' C CRC, careful analysis is required to identify cell lines which are representative models of CRC genomics. © 2001 Cancer Research Campaign http://www.bjcancer.com

**Keywords**: CGH; colo-rectal tumours; cell lines; survival; cancer genomics

Colorectal cancer (CRC) is the third leading cause of cancer deaths, with an estimated 430 000 deaths per year world-wide (Pisani et al, 1999). A multidisciplinary approach, which includes surgery, chemotherapy and radiotherapy, is taken when treating carcinoma of the large bowel. However, approximately half of the patients who undergo potentially curative surgery for CRC will eventually die from recurrent disease (Boring et al, 1993). Disease stage at presentation is the major predictor of outcome, with early stage tumours (Dukes' A & B) having a much better survival than late stage disease (Dukes' C & D). However, there is considerable variability in survival rates within these classifications (Moertel et al, 1995). Efforts to complement the pathologic staging system with markers of prognosis in CRC are still at a relatively early stage of development and most studies are focused on the correlation of protein expression with eventual outcome (McLeod et al, 1999). This is of particular importance in locally advanced CRC (Dukes' C), where systemic chemotherapy is administered to most patients even though it will improve survival for only 15% of patients (Moertel et al, 1995).

Over the last decade, a substantial amount of knowledge has been accumulated using cytogenetics and molecular analyses to elucidate the mechanisms behind CRC. The current understanding of the genetic events responsible for the stepwise progression from normal colonic epithelium to invasive carcinoma (Fearon and Vogelstein, 1990) has been greatly aided by whole genomic analysis of this tumour type by comparative genomic hybridization (CGH) (Kallioniemi et al, 1992; Rooney et al, 1999). CGH has identified specific chromosomal regions which are consistently gained (e.g. 20q and 13q) or lost (e.g. 18q) at a high frequency in CRC and has demonstrated an increase in the genetic grade of a tumour (i.e. the overall number of genomic aberrations) with disease progression (Reid et al, 1996; Al-Mulla et al, 1999). What is less well defined is the influence of genomic aberrations on patient survival.

In this report, the specific genomic aberrations and overall level of chromosomal stability, as identified by CGH, was evaluated in Dukes' C CRC and assessed for an influence on survival. In addition the chromosomal aberrations identified in the Dukes' C tumours were compared with a panel of 8 CRC cell lines to evaluate their usefulness as model systems for CRC genomics.

### MATERIALS AND METHODS

### Chemicals

DNA polymerase I, DNase I, Igepal CA-630 and dNTPs were obtained from Sigma-Aldrich (Poole, Dorset, UK). SpectrumRed dUTP and SpectrumGreen dUTP were purchased from Vysis (Richmond, Surrey, UK). Human Cotl DNA, RPMI 1640 media, fetal calf serum, glutamine, penicillin and streptomycin were from GibcoBRL (Gaithersburg, MD, USA) and formamide from Fluka (New-Ulm, Germany). The Nucleon II extraction kit used

*Received 6 April 2001*
*Revised 17 July 2001*
*Accepted 1 August 2001*

*Correspondence to:* Dr HL McLeod, Washington University School of Medicine, Department of Medicine, 660 South Euclid Ave, Campus Box 8069, St Louis,MO 63110-1093, USA

was from Scotlab (Coatbridge, UK), dextran sulphate was from Pharmacia Biotech (Uppsala, Sweden), and DAPI/antifade was purchased from Amersham Oncorappligene (Durham, UK). All other reagents, chemicals and buffers were purchased from BDH (Poole, Dorset, UK).

### Cell lines growth conditions

All eight colorectal cell lines (R10, H630, RKO, HT29, DLD1, CACO2, BE, LOVO) were cultured in RPMI 1640 or DMEM/F10 medium supplemented with 10% fetal bovine serum, 2mM L-Glutamine and 100U penicillin/streptomycin in a 37˚C incubator with 5% $CO_2$. All cell lines were passaged once a week using trypsin/EDTA and split 1:10, with a subsequent media change every 3–4 days. The cell lines were maintained in standard media and were not selected for any distinct cellular or molecular phenotype.

### Tumour samples

Primary tumours were collected from 29 consecutive patients undergoing surgery for Dukes'C CRC at Grampian University Hospitals Trust. This included 13 men and 16 women, with a median age at diagnosis of 67 years (range 45–86 years). The 29 tumours studied were from the proximal colon ($n$ = 9), distal colon ($n$ = 16), and rectum ($n$ = 4). The minimum clinical follow-up was 22 months, with a median of 29 months. At the time of resection, a consultant pathologist dissected out a sample of the tumour which was then snap-frozen in liquid nitrogen and stored at –80˚C. Cells were isolated from colorectal tumours as follows: Snap-frozen pieces of tumour were removed from –80˚C storage and placed in a –20˚C cryostat machine. For each tumour, 6 sections were cut: 1–5 µm section and 5–20 µm sections. The 5 µm section was then stained with haematoxylin and used as a guide to remove normal cells from the 20 µm sections. Microdissection was performed with a scalpel using a dissecting light microscope with a 20X objective. Following this, all the remaining cells (tumour cells) were scraped into a 1.5 ml microfuge tube containing reagent A from a Nucleon II DNA extraction kit (Coatbridge, UK). DNA extraction was performed according to the manufacturer's instructions. Only tumour specimens with over 50% tumour cellularity were included for CGH analysis. The concentration of tumour DNA was quantified using a fluorimeter.

### Comparative Genomic Hybridization

CGH was performed as previously described (Rooney et al, 1998). As a negative control, 200 ng of healthy human DNA labelled with SpectrumGreen was hybridized with 200 ng of the same DNA labelled with SpectrumRed fluorochrome. In addition, to confirm equal sensitivity of both fluorochromes for detection of gain and loss, and to reduce the likelihood of false positives (Barth et al, 2000), all experiments were repeated with reverse labelling of DNA (i.e. tumour DNA was labelled with SpectrumRed dUTP and the reference DNA with SpectrumGreen dUTP). DAPI counterstain was used to karyotype all processed metaphase spreads. For each competitive hybridization reaction at least 10 metaphase spreads were analyszed. Chromosomal regions were interpreted as over-represented (gain) in the test DNA if a fluorescence ratio (FR) of green to red fluorescence of $> 1.15$ was observed, while regions with a FR of $< 0.85$ were interpreted as under-represented (loss). Any region with a FR $> 1.5$ indicated high level amplification. Genetic grade was calculated as the sum of the number of losses and gains found in the tumour.

### Statistical analysis

Kaplan-Meier log-rank analysis was used to evaluate the influence of both specific chromosomal aberrations and the overall genetic grade of each tumour on patient survival. A probability value of less than 0.05 was considered significant. Statistical analyses was carried out using SPSS for Windows 95 version 9.0 (SPSS UK Ltd, Woking, Surrey, UK).

### RESULTS

A high level of variation was seen in both the number and type of genetic aberrations detected in the 29 Dukes' C colorectal tumours and the 8 colorectal cell lines assessed by CGH (Tables 1 and 2).

**Table 1** Chromosomal aberrations detected by CGH in 8 colo-rectal cancer cell lines

| Cell line | No. of Gains | No. of losses | Total no. of aberrations | CGH aberrations (+ = Gain, – = Loss and ++ = High level amplification) |
|---|---|---|---|---|
| R10 | 9 | 6 | 15 | +1q21, +2p16-pter, +5p, +9p, +10p, +11q13-qter, +13q, +15q22–25, +20q12–13.2, –1p, –3p14–3q13.1, –4q, –5q -7p22 & –18q |
| H630 | 8 | 6 | 14 | +2p13–23, +5p, +7p11.23, +9p21–22, +10p12-pter, ++13q, +18p, +20, –1p22.2–31.1, –4q, –5q15–31, –9q33-qter, –10q25 & –18q12.3-qter. |
| RKO | 2 | 2 | 4 | +7q32–34, +8q21.1-qter, –2q37 & –6p23-pter |
| HT29 | 5 | 4 | 9 | ++8q22-qter, +11, +12q24.1-qter, +13q12.2, +20q, –3p12.3–13, –8p21-pter, –18q & –21q |
| DLD1 | 2 | 2 | 4 | +1p35-pter, +9q34-qter, –13q31 & –18 |
| CACO2 | 6 | 3 | 9 | +10q21-pter, +11q13-qter, +12p, +16q23-qter, +17q +20q, –1p, –9p22-pter & –18q |
| BE | 3 | 4 | 7 | +14q22-qter, +19q ++20q, –8q21.2-pter, –9p21-pter, –10p15 & –18q21-qter |
| LOVO | 1 | 0 | 1 | +12q24.1-pter |

*© 2001 Cancer Research Campaign* 

**Table 2** Chromosomal aberrations detected by CGH in 29 Dukes' C colo-rectal tumours

| Tumour no. | Status (alive [1] dead [0]) | Follow-up (months) | CGH aberrations (+ = Gain, – = Loss and ++ = High level amplification) |
|---|---|---|---|
| 1 | 1 | 54 | +8p22–23, +8q22–24.2, +11p15, +20p, –2q21, –4q34–35, –6p, –6q, –9cent-q13, –10p, –10q, –12cent-q13, –13q21 & –14q32.3 |
| 2 | 0 | 28 | +5p21, +6p25, +7p, +7q, +10p15, +20p12, +20q12-qter, –1q12, –9q12–21, –12q24.1-qter, –13q21.2-qter, –16p11.2-16q11.2, –18cent-p11.2, –19p & –19q |
| 3 | 0 | 14 | +8q22-qter |
| 4 | 1 | 49 | none detected |
| 5 | 1 | 29 | –2p, –2q, –8p12–21, –10q22-qter, –15q22 & –18q21–23 |
| 6 | 1 | 25 | +1q31–32, +3p26.1, +8q, +13q, +20p, +20q & –1p36.1, –3p22-qter, –4p, –9p, –16p13.2–13.3, –18p, –18q & –22q13 |
| 7 | 1 | 24 | +1p33–1q21 & –9p24-q21 |
| 8 | 0 | 8 | –3p25–26 |
| 9 | 1 | 23 | +1p12–q31, +1q12–22, +2p12–12, +5p, +7p, +13q32, +14q12–13, –3cent-q21, –4p12–14, –14q32, –17p13, –17q11.2, & –18p11.3 |
| 10 | 1 | 23 | +1q12–21,+8q, +9q12–21,+20p, +20q, –2q23, –4q22–24, –5q15–31,–6q16-qter, –8p & –18q12–22 |
| 11 | 1 | 35 | +1q12, +5cent, +13q, +16p11.2-q21.2, +16p13.3, +20q, –1q, –2p, –7p13–21, –9p12, –15q13–21, –18q12 & –21q22 |
| 12 | 1 | 28 | none detected |
| 13 | 0 | 19 | none detected |
| 14 | 1 | 38 | +1p12–21,+7p, +7q, +13q, +16p, +16q, ++20q, –1p13–31, –3q11.2–13.1,–5q13–23, –8p23, –17p, –18p, –18q, –21q22 |
| 15 | 1 | 28 | +2p16, +2q21–24, +4p12–14, +4p15, 4q22, +7p13–15, +7q31, +8q23–24.1,+9p22, +13q, +14p15, +16p13.1-qter, +16q23-qter, –1q12, –4p16, –8p21–22, –17p, –17q & –19p |
| 16 | 1 | 50 | –9p22–24 |
| 17 | 1 | 43 | +6p25, +17q22–24, –4q21–22 & –11p13–14 |
| 18 | 1 | 46 | none detected |
| 19 | 1 | 22 | +8q22–24.1,+12p13, +13q, +20q, –7q22–31, –14q, –15q24-25, –17p11.2, –18p, –18q & –20p |
| 20 | 0 | 13 | –15q & –17p12–13 |
| 21 | 1 | 45 | +3q23–25, +5q14–21, +17p13, +20q13.2–13.3, & –14q23–24 |
| 22 | 1 | 29 | none detected |
| 23 | 1 | 29 | +2q24–32, +7p, +12p, +12q, +13q, +20p, +20q, –4q27-qter, –6q21-qter, –11q13-qter, –18p, –18q & –21q |
| 24 | 0 | 7 | none detected |
| 25 | 0 | 12 | –19q13.2–13.4 |
| 26 | 1 | 47 | +9q12–21.2, –19p13.2 & –21q22 |
| 27 | 0 | 43 | +7p21–22, +7q21, +9p24, +17p13, –2q, –4q33–34, –11p14–15 & –15q13–14 |
| 28 | 1 | 29 | +2p, +3p22, +7p, +7q, +11q23, ++13q, +19q12–q13.1,+20p, –1p, –4p, –4q, –5p, –5q, –10p, –10q, –12p, –12q, –15q24–q25, –17p & –18q |
| 29 | 0 | 6 | –16q24 & –18q12–22 |

Almost every chromosomal arm was detected as changed in at least 1 of the 37 cancer genomes assessed (Figures 1 and 2).

Genome-wide analysis of some tumours (i.e., tumours 4, 12, 13, 18, 22, 24) detected no changes, while other cases showed multiple aberrations (e.g., tumours 15 and 28 showed a total of 17 and 20 chromosomal aberrations, respectively) (Table 2). The median number of gains per tumour was 2.0 (mean 3.7, 108 gains/29 tumours), while the median loss was 1.0 aberration per tumour (mean 2.9, 85 losses/29 tumours). The median genetic grade was 4.0 (mean 6.7, 193 aberrations/29 tumours). For tumours the genetic grade was not normally distributed, with the suggestion of a multimodal distribution (Figure 4). The chromosomal regions most frequently gained in the 29 tumours globally assessed by CGH were 13q (27.6%), 20q (27.6%), 7p (24.1%), 8q (24.1%) and 1q (20.7%) (Table 2; Figure 3). The most frequent losses of chromosomal material in the tumours occurred at 18q (31%), 4q (20.7%), 17p (20.7%), 18p (20.7%) and 15q (20.1%) (Table 2; Figure 3). High level amplification (FR > 1.5) was seen in two

 *© 2001 Cancer Research Campaign*

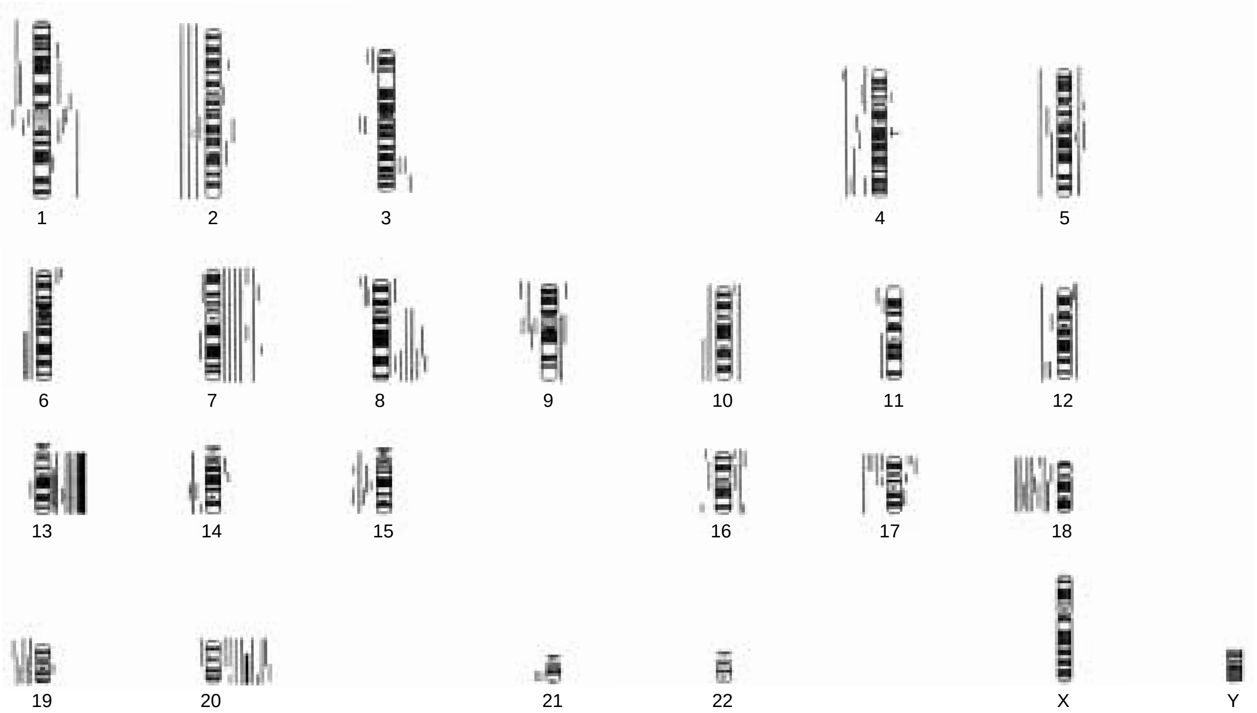

**Figure 1** Summary of regions of genomic change in tumour DNA from 29 Dukes' C colorectal tumours detected by CGH. Bars on the left side of the chromosome ideogram denote a loss, bars on the right side a gain of sequence in the tumour genome. Regions of high-level copy number increases (amplifications) are indicated by solid/bold bars

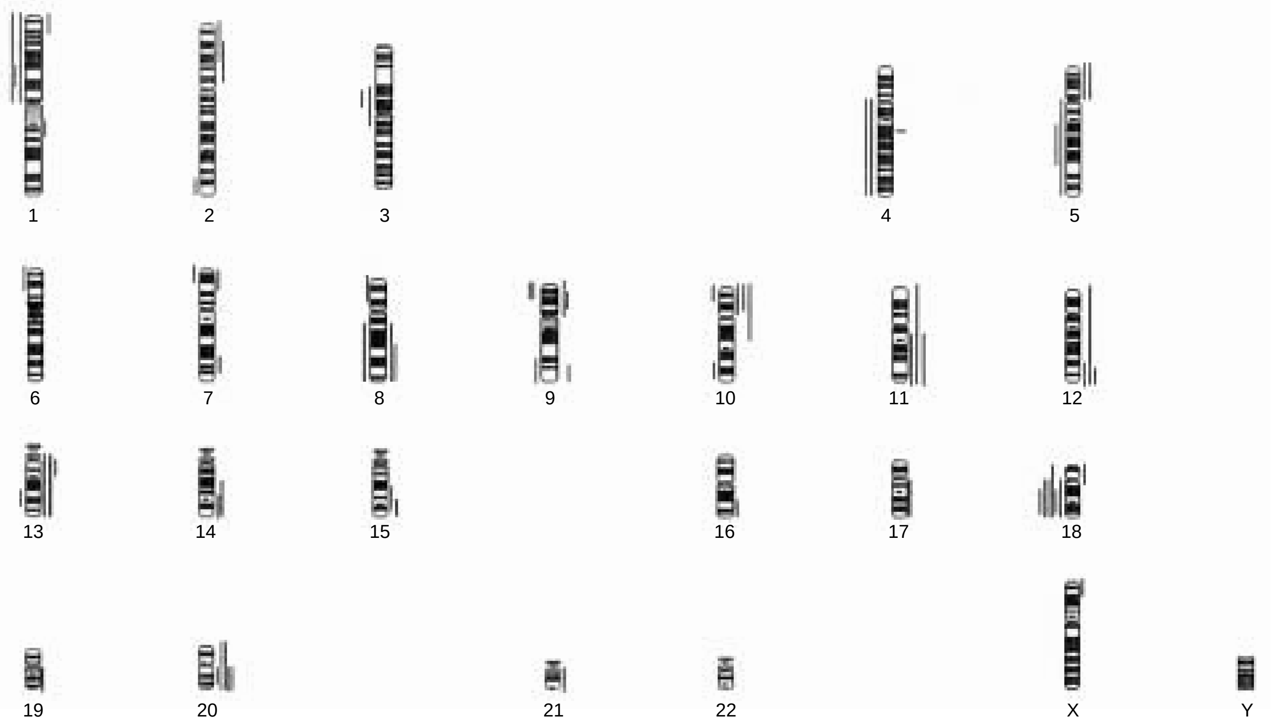

**Figure 2** Summary of regions of genomic change in DNA from 8 colo-rectal cancer cell lines detected by CGH. Bars on the left side of the chromosome ideogram denote a loss, bars on the right side a gain of sequence in the tumour genome. No regions of high-level copy number increases (amplifications) were detected tumours; tumour 14 on 20q (whole arm) and tumour 28 on 13q (whole arm).

In the cell lines gain of chromosomal material was slightly more common than loss (mean 4.5 gains, 36 gains/8 cell lines; mean 3.4 losses, 27 losses/8 cell lines). The median number of gains per cell line was 4.0 while the median number of losses was 3.5. The median genetic grade was 8.0 (mean 7.8, 63 aberrations/8 cell lines). 20q was the most common region of gain, being detected in

© 2001 Cancer Research Campaign

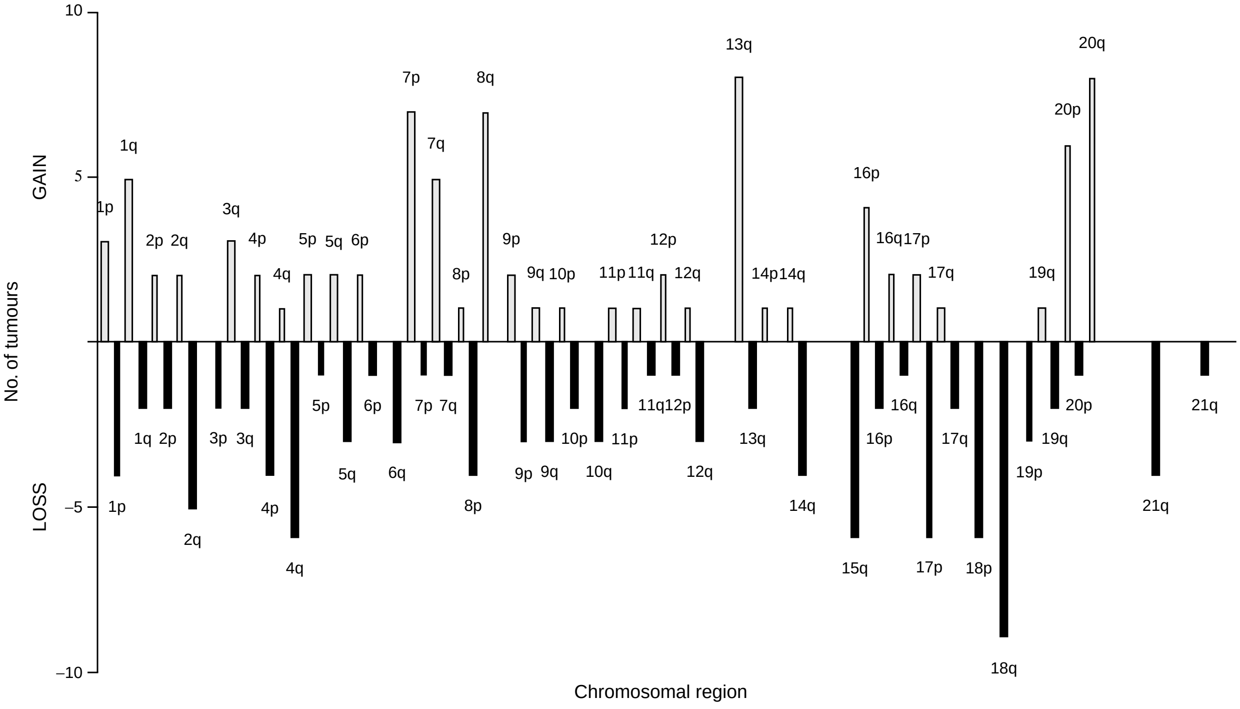

**Figure 3** The observed regions of loss and gain detected by CGH analysis among 29 colo-rectal tumours

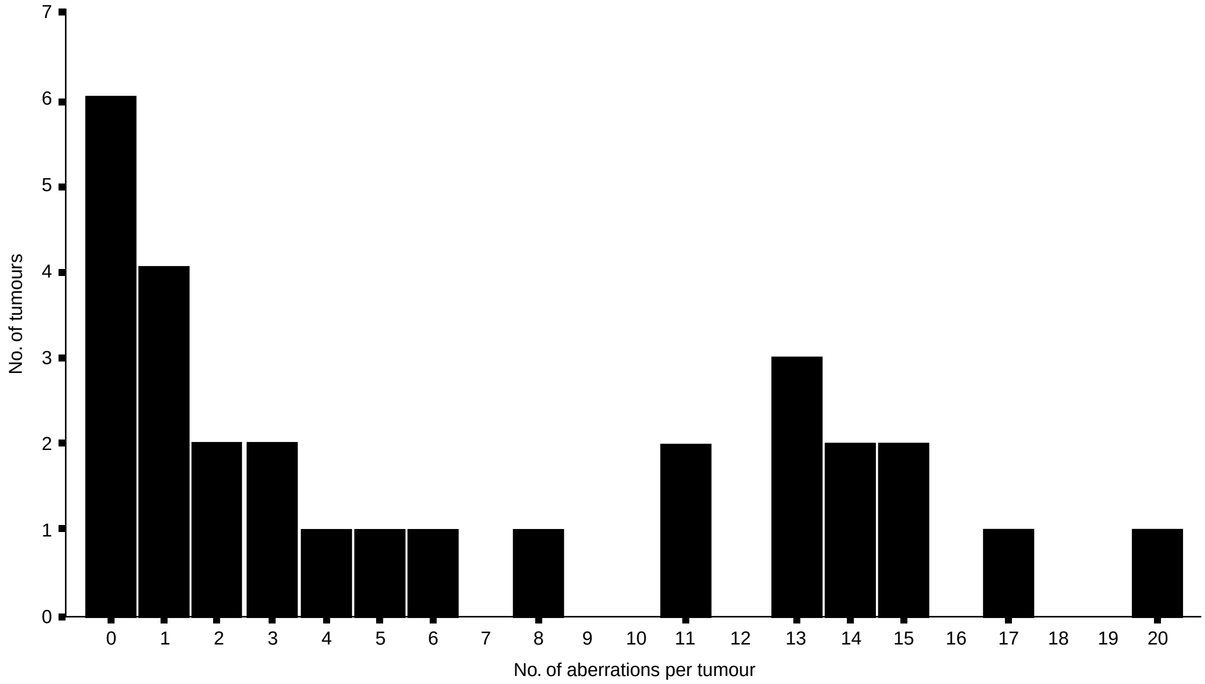

**Figure 4** Frequency distribution of the genetic grade (number of aberrations) in 29 colo-rectal tumours

62.5% (5/8 cell lines) of all the cell lines assessed. Other common regions of gain included; 11q and 13q which were both gained in 37.5% or 3/8 of cell lines. Considering the chromosomal arms most commonly lost across the cell lines, 18q showed the highest level of aberration being detected in 75% of the cell lines (6/8). 1q was the second most commonly lost region, found changed in 37.5% of the cell lines (3/8). All cell lines contained at least one aberration detectable by CGH.

The 5 most common regions of gain (13q, 20q, 7p, 8q, 20p) and the 5 most common losses (18q, 4q, 15q, 17p, 18p) in the 29 CRC tumours were evaluated for influences on patient survival. No association between any specific chromosomal aberration and patient survival was demonstrated ($P > 0.05$ for all analysis). However, the level of chromosomal aberration detected per tumour (the genetic grade) was found to be associated with survival. Patients with tumours containing > 2 aberrations had a

 *© 2001 Cancer Research Campaign*

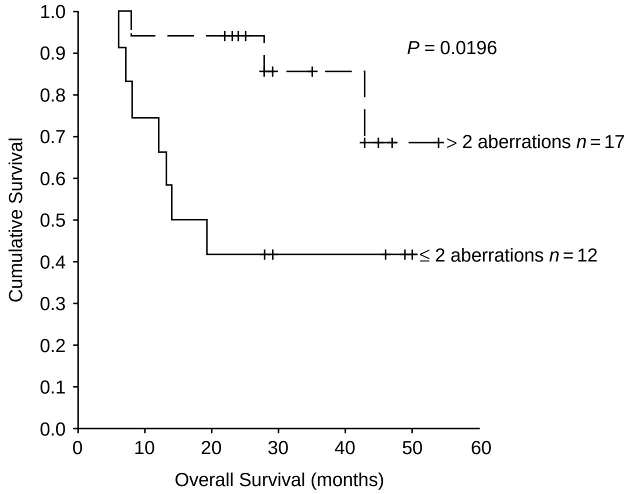

**Figure 5** Influence of chromosome instability on survival in patients with Dukes' C colo-rectal cancer, as detected by CGH

better survival than those with tumours containing ≤ 2 aberrations ($P$ = 0.02, Figure 5). 95% of patients with > 2 aberrations (16/17) were alive at 22 months (the minimum follow-up for all patients) compared to 42% of patients with tumours displaying ≤ 2 aberrations (5/12). The median survival was 14 months in patients with ≤ 2 aberrations, where as median survival was not yet reached in the patients with > 2 aberrations per tumour.

### DISCUSSION

In this analysis of Dukes' C CRC, a significant difference in survival was observed between tumours which had > 2 aberrations and those which had ≤ 2 aberrations. In contrast, analysis of the top five gains and losses detected by CGH found no association between specific chromosomal aberrations and survival. This data suggests that the overall genomic context of the tumour, rather than the specific aberrant loci, is of clinical relevance in CRC. While this varies from the traditional dogma that specific genes (e.g., angiogenic proteins, metalloproteinases, antiapoptotic genes, therapeutic targets) are mediating the outcome of advanced cancer, the current findings are in line with the recent emphasis on genomic instability as a key event in tumour cells (Cahill et al, 1999). The mechanistic basis for altered genetic grade in CRC is not clear, but mutations in several candidate pathways (e.g., mitotic spindle checkpoint) have been identified (Cahill et al, 1998).

To date, no other study of CRC has attempted to associate tumour chromosomal stability/genetic grade with survival, as detected by CGH. However, a recent study investigating a less common mechanism for a mutator phenotype, microsatellite instability (MIN), also detected an association between genetic stability and survival (Gryfe et al, 2000). Analysis of clinical outcome in both unselected CRC patients and those ≤ 50 years of age found that MIN was independently predictive of a favourable outcome. Why increased genetic instability, whether it manifests itself as MIN (Gryfe et al, 2000) or increased chromosomal aberration (as detected here), should be associated with a favourable outcome is not known. It has been suggested that a tumour can have an excess of genomic instability, spurring the tumour cells into various mechanisms of cell death (Cahill et al, 1998).

CGH analysis identified several regions of the genome associated with tumourigenesis in the Dukes' C colorectal tumours. The general trend of genomic instability detected in the carcinomas assessed in this study mirrors that identified by CGH in other colorectal carcinomas (Rooney et al, 1999). Although the trend of loss and gain is similar, there is variation between the observed frequency of specific altered regions between the current study and the pooled data from previous reports. For example, gain of chromosome 13q was detected in 27.6% of carcinomas in this study, but in 50% in other studies of colorectal carcinoma by CGH (Reid et al, 1996; De Angelis et al, 1999; Georgiades et al, 1999). This could reflect the focus on a single disease stage (Dukes' C). However, there is a more likely technical explanation for these differences. In this study, only aberrations detected in both the forward and reverse CGH reaction were used in the analysis. The addition of a fluorochrome reversal step to conventional CGH reduces the risk of false positives, a potential problem with many currently used CGH protocols (Barth et al, 2000). It also allows the sub-chromosomal regions identified as altered by CGH to be more precisely defined by using two profiles to define a region of change rather than one. If the approach used in this study is more thorough, then this data suggests that the CGH literature may be over-estimating the level of genetic aberrations in CRC tumours.

The genetic grade for the Dukes' C CRC was not unimodal (Figure 4). Six tumours (21%) had no aberrations detected, 12 (41%) had between 1 and 8 aberrations detected per genome assessed, while the other group of tumours had between 11 and 20 (38%) aberrations per tumour. A similar distribution was recently observed in a study of CRC using CGH (De Angelis et al, 1999). This multimodal distribution of chromosomal aberrations across CRC tumours is consistent with the hypothesis that multiple phenotypes exist for CRC (Georgiades et al, 1999; Hawkins et al, 2001).

The value of cell lines to further study of genetic aberrations observed by CGH analysis has already been demonstrated in breast cancer. In this tumour type initial detection of a region of amplification on chromosome arm 20q followed by high density fluorescence in situ hybridization mapping experiments in breast cancer cell lines helped isolate several putative oncogenes within the amplicon (Kallionemi et al, 1994; Collins et al, 1998). Adapting a similar approach to the study of genomic aberrations associated with CRC should speed the identification of the crucial genes driving the CGH signature. There is concern about the relevance of in vitro model systems, as previous studies have demonstrated that continuous culture of tumour cell lines allows, and possibly encourages, random genomic aberrations (Reiss et al, 1986; Jones et al, 2000). Comparing the CGH data for the 29 solid tumours and 8 cell lines in this study found that the regions of alteration were similar in both systems. Furthermore, the most common aberrations were often the same for both systems. For example, 18q loss and 20q gain were the most common region of loss and gain in both the tumours and the cell lines. However, the actual frequency of the aberrations was higher in the cell lines. For example, 20q gain was detected in 27.6% of the tumours and 62.5% of the cell lines, while 18q was detected as lost in 31% of tumours and 75% of cell lines. The median number of total aberrations per cell line was 8 compared to a median of 4 for the colorectal tumours. Furthermore, 21% of CRC had no aberrations, while 100% of cell lines had at least one region of loss or gain. These results support the recent hypothesis that some colorectal genotypes (particularly diploid tumours) could be absent in currently available cell culture models (Hawkins et al, 2001). The mechanistic basis for the observed bias in genome aberration frequency is not yet defined.

© 2001 Cancer Research Campaign

The results of this study suggest that above a certain threshold of genomic instability (e.g., > 2 aberrations), an unstable genome confers a survival advantage in CRC. Whether this threshold associated with survival is directly related to tumour aggression or is specifically conferring resistance to therapies, such as chemotherapy, is not known. In addition, the chromosomal aberrations detected by CGH analysis of colo-rectal solid tumours and colo-rectal cell lines were present in both systems, but the frequency of aberrations was greater in the cell lines. This suggests that many cell lines would be appropriate models for the subset of tumours with high frequency chromosomal changes, but careful evaluation is required to identify those representative of most Dukes' C CRC.

### ACKNOWLEDGEMENTS

This work was supported in part by the Scottish Hospital Endowment Research Trust (SHERT) and the Aberdeen Colorectal Cancer Initiative (Steering committee Jim Cassidy, Howard L. McLeod, Graeme I. Murray, Neva E. Haites, Julian Little, William T. Melvin).

### REFERENCES

Al-Mulla F, Keith NW, Pickford IR, Going J and Birnie GD (1999) Comparative genomic hybridisation analysis of primary colorectal carcinomas and their synchronous metastases. *Gene Chromosome Cancer* **24**: 306–314

Barth TFE, Benner A, Bentz M, Döhner H, Möller P and Lichter P (2000) Risk of false positive results in comparative genomic hybridisation. Gene Chromosome *Cancer* **28**: 353–357

Boring CC, Squires TS and Tong T (1993) Cancer Statistics. *Cancer J Clin* **43**: 7–10

Cahill DP, Lengauer C, Yu J, Riggins GJ, Wilson JK, Markowitz SD, Kinzler KW and Vogelstein B (1998) Mutations of mitotic checkpoint genes in human cancers. *Nature* **392**: 300–303

Cahill DP, Kinzler KW, Vogelstein B and Lengauer C (1999) Genetic instability and Darwinian selection in tumours. *Trends Cell Biol* **9**: M57–60

Collins C, Rommens JM, Kowbel D, Godfrey T, Tanner M, Hwang S, Polikoff D, Nonet G, Cochran J, Myambo K, Jay KE, Froula J, Cloutier T, Kuo WL, Yaswen P, Dairkee S, Giovanola J, Huechison BG, Isola J, Kallioniemi OP, Palazzolo M, Martin C, Ericsson C, Pinkel D, Albertson D, Li WB and Gray JW (1998) Positional cloning of ZNF217 and NABC1: Genes amplified at 20q13.2 and overexpressed in breast carcinoma. *Proc Natl Acad Sci USA* **95**: 8703–8708

De Angelis PM, Clausen OPF, Schjolberg A and Stokke T (1999) Chromosomal gains and losses in primary colorectal carcinomas detected by CGH and their associations with tumour DNA ploidy, genotypes and phenotypes. *Br J Cancer* **80**: 526–535

Fearon ER and Vogelstein B (1990) A genetic model for colorectal tumourigenesis. *Cell* **61**: 759–767

Georgiades JB, Curtis LJ, Morris RM, Bird CC and Wyllie AH (1999) Heterogeneity studies identify a subset of sporadic cancers without evidence for chromosomal or microsatellite instability. *Oncogene* **18**: 7933–7940

Gryfe R, Kim H, Hsieh ET, Aronson MD, Holowaty EJ, Bull SB, Redston M and Gallinger S (2000) Tumour microsatellite instability and clinical outcome in young patients. *N Engl J Med* **42**: 69–77

Hawkins NJ, Tomlinson I, Meagher A and Ward RL (2001) Microsatellite-stable diploid carcinoma: in a biologically distinct and aggressive subset of sporadic colorectal cancer. *Br J Cancer* **84**: 232–236

Jones C, Payne J, Wells D, Delhanty JDA, Lakhani SR and Kortenkamp A (2000) Comparative genomic hybridization reveals extensive variation among different MCF-7 cell stocks. *Cancer Genet Cytogen* **117**: 153–158

Kallioniemi A, Kallioniemi OP, Piper J, Tanner M, Stokke T, Chen L, Smith HS, Pinkel D, Gray JW and Waldman FM (1994) Detection and mapping of amplified DNA-sequences in breast-cancer by comparative genomic hybridization. *Proc Natl Acad Sci USA* **91**: 2156–2160

Kallioniemi A, Kallioniemi O-P, Sudar D, Rutovitz D, Gray JW, Waldman F and Pinkel D (1992) Comparative genomic hybridisation for the genetic analysis of solid tumours. *Science* **258**: 818–821

McLeod HL and Murray GI (1999) Tumour markers of prognosis in colorectal cancer. *Br J Cancer* **79**: 191–203

Moertel CG, Fleming TR, MacDonald JS, Haller DG, Laurie JA, Tangen CM, Ungerleider JS, Emerson WA, Tormey DC, Glick JH, Veeder MH and Mailliard JA (1995) Fluorouracil plus levamisole as effective adjuvant therapy after resection of stage-III colon-carcinoma. *Ann Intern Med* **122**: 321–326

Pisani P, Parkin MD, Bray F and Ferlay J (1999) Estimates of the worldwide mortality from 25 cancers in 1990. *Int J Cancer* **83**: 18–29

Reid T, Knutzen R, Steinbeck R, Blegen H, Schröck E, Heselmeyer K, du Manoir S and Auer G (1996) Comparative genomic hybridisation reveals a specific pattern of chromosomal gains and losses during the genesis of colorectal tumours. *Gene Chromosome Cancer* **15**: 234–245

Reiss M, Gambavitalo C and Sartorelli AC (1986) Induction of tumor-cell differentiation as a therapeutic approach-preclinical models for hematopoietic and solid neoplasms. *Cancer Treat Rep* **70**: 201–218

Rooney PH, Stevenson DAJ, Marsh S, Johnston PG, Haites NE, Cassidy J and McLeod HL (1998) CGH analysis of chromosomal alterations induced by the development of resistance to TS inhibitors. *Cancer Res* **58**: 5042–5045

Rooney PH, Murray GI, Stevenson DAJ, Haites NE, Cassidy J and McLeod HL (1999) Comparative genomic hybridisation and chromosomal instability in solid tumours. *Br J Cancer* **80**: 862–873

*© 2001 Cancer Research Campaign*
